# “They take care of their own”: healthcare professionals’ constructions of Sami persons with dementia and their families’ reluctance to seek and accept help through attributions to multiple contexts

**DOI:** 10.1080/22423982.2017.1328962

**Published:** 2017-06-06

**Authors:** Bodil Hansen Blix, Torunn Hamran

**Affiliations:** ^a^ Centre for Care Research, north, Department of Health and Care Sciences, UiT The Arctic University of Norway, Tromso, Norway

**Keywords:** Sami, indigenous, healthcare services, family caregivers, barriers, dementia, equity in healthcare, discourse, narrative context analysis, attribution

## Abstract

**Background**: Norwegian government white papers have stated that the Sami population is reluctant to seek help from healthcare services and has traditions of self-help and the use of local networks.

**Objective**: In this article we explore healthcare professionals’ discursive constructions of Sami persons with dementia and their families’ reluctance to seek and accept help from healthcare services.

**Design**: The article is based on an analysis of focus group interviews with healthcare professionals (n = 18) in four municipalities in Northern Norway with multiethnic populations. A narrative context analysis, which involved an examination of sequences of discourse, was employed.

**Results**: Reluctance to seek and accept help among Sami service users and assumptions about self-support were recurring themes in the focus groups. The reluctance was attributed to macro contexts, such as socio-historical processes and cultural norms, and to micro contexts, such as individual and interpersonal factors including the healthcare professionals’ cultural backgrounds and language competence. The healthcare professionals’ positioning as insiders or outsiders (Sami or non-Sami) affected their attributions.

**Conclusions**: Local healthcare professionals are at the front line for providing and assessing service users’ needs for healthcare services. Consequently, their perceptions of service users’ needs are pivotal for achieving equity in healthcare. The established opinion that Sami “take care of their own” and are reluctant to seek and accept help may lead to omissions or neglect. Healthcare professionals’ awareness about how present encounters in healthcare settings are framed and shaped by the service users’ previous and prevailing experiences of marginalisation and subordination is crucial to avoid omissions or neglect resulting from assumptions about cultural preferences. Discursively shaped boundaries and differences between groups may create the impression that the distance between the groups is too wide to traverse, which in turn may lead to further marginalisation of service users in healthcare encounters.

In Norway, the Sami people’s right to safeguard, preserve and develop their language, culture and way of life is stated in both the Norwegian Constitution and the Sami Act [1,2]. The rights of the Sami people with regard to healthcare are related to more general legislation, such as the Patients’ Rights Act [3]. The healthcare rights of the Sami people are also based on international conventions, such as The United Nations (UN) Declaration on the Rights of Indigenous Peoples [4] and The UN International Covenant on Civil and Political Rights [5], which is incorporated into Norwegian legislation through the Human Rights Act [6]. The International Labour Organisation (ILO) Convention No. 169 concerning Indigenous and Tribal Peoples [7] states that health services should be community based to the greatest extent possible and should be planned and administered in cooperation with the community served. Furthermore, the importance of training and employing local community health workers is emphasised.

The Norwegian official policy is to “integrate the Sami perspectives in the ordinary care services” rather than to develop care services exclusively for the Sami population [8,9]. However, the meaning of “Sami perspectives” remains unclear. The level of satisfaction regarding healthcare services in Norway is generally high. However, Sami individuals feel their needs are “not being met with the necessary respect for their values and preferences” and that information is not available “in a form or in a language that renders possible active involvement in the design of the services” [9, p. 50]. Several government white papers have stated that the Sami population is reluctant to seek help from public health and care services [10–12]. Furthermore, it has repeatedly been stated that the Sami have strong traditions of self-help and the use of local networks [9–12].

In this article, we explore healthcare professionals’ perceptions of the use and non-use of local public healthcare services by Sami persons with dementia and their families (in this article described as “service users”). Specifically, we inquire into their narrations about service users’ reluctance or refusal to accept help from public healthcare services and how this reluctance is attributed to various contexts.

## Background

### The Sami

The Sami are indigenous people living in Norway, Sweden, Finland and the Russian Kola peninsula. The Sami population is estimated to range from 50,000 to 80,000 individuals [13], but the vast majority of Sami reside in Norway, where the Sami population is estimated at 40,000 [14,15]. In Norway, approximately 25,000 Sami speak a Sami language [16]. National governments have made strong efforts to assimilate the Sami into the majority populations, but the assimilation process has been paralleled by individual experiences of stigmatisation, discrimination and “everyday racism” [17]. Defining who the Sami are is not a straightforward task. The history of the public assimilation policy, the co-existence of several ethnic groups (i.e. Sami, Norwegians and Kvens, the descendants of the Finnish-speaking minority in Norway) in the same geographic area [18], and the history of interaction and intermarriage between ethnic groups [19] have resulted in a complex ethnic situation.

### Previous research

Research has not unambiguously supported the assumption that the Sami use healthcare services to a lesser extent than the majority population. For example, Norum and Nieder [20] found that inhabitants of Sami-speaking municipalities were referred to specialist healthcare services as frequently as people living in control municipalities. However, these authors found large inter-municipal variations in both groups. Gaski et al. [21] concluded that no ethnic barriers to the utilisation of somatic hospital and specialist services existed, based on findings that overall expenditures on these services in Sami municipalities were above the national average and equivalent to corresponding municipalities in the same geographic area [21]. However, neither of these studies addressed the quality of healthcare as experienced by the Sami. A study by Nystad et al. [22] indicated that Sami-speaking patients are less satisfied than other patients with services provided by municipal general practitioners [22], and a study of mental healthcare found that Sami patients were less satisfied with treatment, contact with staff, and treatment alliance than Norwegian patients [23]. Moreover, research on the experiences of bereaved people after traumatic deaths among the Sami has nuanced the assumption that the Sami are, or prefer to be, self-supported [24]. The bereaved stressed that although support from family and friends was of great importance, it was not sufficient and asked for “active outreach” from healthcare professionals at an early stage.

There are no estimates of the prevalence of dementia in the Sami population. The condition is, however, largely underdiagnosed in the overall population. Contact with healthcare services is crucial to being examined and diagnosed, and diagnosis is crucial to gaining access to care. To our knowledge, no prior research has focused on the use and non-use of healthcare services among Sami persons with dementia and their families. However, research has demonstrated that people with dementia from other minority ethnic groups tend to present to care services later, and when their illness is more severe, than majority populations [25–27]. A systematic review of studies on help seeking for dementia among minority ethnic groups identified barriers such as negative experiences, discrimination, language barriers, uncertainty regarding where and how to access help, lack of knowledge about dementia and the belief that nothing could be done to help [27]. Research with minority ethnic family caregivers has revealed that the sense of familial responsibility and negative perceptions of psychiatric services are important barriers to early contact with healthcare services [26]. A recent study focusing on the collaboration between formal and family caregivers in Northern Norway demonstrated that collaboration was hampered by ethnic and ethno-political positions [28].

Local healthcare professionals are at the front line in providing and assessing service users’ needs for care services. Consequently, their perceptions of service users’ needs are pivotal for achieving equity in healthcare. To our knowledge, no previous research has inquired into the use and non-use of care services by Sami people with dementia and their families from the perspective of healthcare professionals.

## Methods

This article is based on analysis of focus group interviews with healthcare professionals from four Northern Norway municipalities that have both Sami and Norwegian populations. The interviews were conducted within the framework of a larger study that focused on cooperation between formal and informal caregivers of persons with dementia.

### Participants and recruitment

The participants (n = 18) in the four focus groups were recruited from four Norwegian municipalities, all of which are included in the administration area of the Sami language law. The only inclusion criterion was that participants were involved in providing everyday care for users of local healthcare services, which means they were either registered nurses (RN) or licensed practical nurses (LPN). The focus groups are presented in Table 1. Although we did not request such information, the participants’ self-identifications as Sami or non-Sami were often revealed during the interviews. Their work experience ranged from seven to 40 years in the public healthcare service sector. All the participants were women, which reflects the fact that the majority of RNs and LPNs in local healthcare services for older adults in Norway are women. The managers of local care services distributed informational material and consent forms to potential participants, and signed consent forms were returned directly to the researchers in prepaid envelopes. Consequently, local managers had no information about who chose to participate in the study. After receiving letters of consent, focus group interviews were scheduled in the respective communities. Focus groups varied in size and composition, with the smallest consisting of only two participants and the largest consisting of eight.

**Table 1.** Focus groups.

Focus group	Number of participants	Participants’ professions	Interview duration (min.)	Identification used in the text
FG1	8	RN: 5 LPN: 3	90	1–1, 1–2, 1–3, 1–4, 1–5, 1–6, 1–7, 1–8
FG2	2	RN: 1 LPN: 1	60	2–1, 2–2
FG3	5	LPN: 5	105	3–1, 3–2, 3–3, 3–4, 3–5
FG4	3	RN: 3	110	4–1, 4–2, 4–3

### Focus group interviews

Our understanding of a focus group is consistent with Barbour [29, p. 2], who noted that “any group discussion may be called a focus group as long as the researcher is actively encouraging of, and attentive to, the group interaction […] ensuring that participants talk amongst themselves rather than interacting only with the researcher”. In our study, we were interested in how understandings and attributions were constructed and negotiated among the healthcare professionals. Hence, focus groups were a suitable approach.

Focus groups were conducted in meeting rooms at local nursing homes or health centres, and were digitally recorded. Two researchers were present during the interviews; one was responsible for asking questions and initiating group discussions, and the other focused on observing and taking notes regarding group interactions and identifying new leads as they appeared in conversations.

A broad topic guide was used in the interviews. It included topics such as descriptions of the districts, descriptions of the service users (e.g. geographic distribution, age span, networks), the establishment of contact between the service users and the healthcare services, experiences of offered services being rejected, experiences of requested services not being available, collaboration with families and other informal caregivers, and the distribution of responsibilities between informal caregivers and the healthcare services. Immediately after the interviews, researchers discussed the interviews and wrote field notes; interviews were then transcribed.

### Ethics

The study was approved by the Norwegian Social Science Data Services. All participants provided informed consent to participate. Participants were informed of their right to withdraw from the study without stating a reason, and were assured that confidentiality would be maintained. At the beginning of the interviews, the interviewer described the purpose of the interview and assured the participants of their anonymity. All group members agreed to maintain confidentiality.

### Analysis

Focus group audio recordings were replayed, and the transcribed texts reread several times. The research team examined one interview at a time in a process that involved a purposeful search for segments related to the use and non-use of healthcare services by the Sami. A narrative context analysis, as suggested by De Fina [30], was a suitable approach to study how the healthcare professionals’ narrations about Sami persons with dementia and their families’ reluctance to seek and accept help drew on contexts, shared ideologies and stereotypes about social categories of belonging. De Fina [30, p. 423] noted that:

a link between local meaning-making activities and macro social processes can be found in the negotiation, at the local level and within the constraints of local practices, of the position and roles of the ethnic group in the wider social space.

Furthermore, she argued that there are elements that connect narrations at the micro level (in specific contexts, e.g. in a focus group interview) to aspects of the macro context, such as the status of power relations among ethnic groups [30]. We also draw on Edwards and Potter’s [31] understanding of attributions as something people do rather than something people perceive or think [31]. Consequently, a close examination of sequences of discourse is necessary to understand how such attributions are performed in, and through, language.

With the aid of excerpts from the interview material, we demonstrate that both reluctance to seek and accept help among Sami service users and assumptions about self-support in Sami communities and families were recurring themes in the focus group interviews. Furthermore, we demonstrate that healthcare professionals relate to micro and macro contexts while attributing the Sami service users’ reluctance to seek and accept help to socio-historical, cultural and individual/interpersonal factors. Moreover, we demonstrate that healthcare professionals’ positions as either insiders or outsiders (Sami or non-Sami) affects their discursive attributions.

## Results

### Self-support and reluctance to seek help

The focus group discussions typically started with the healthcare professionals describing the districts they served. The home care services were described as equally available to all inhabitants in the districts, and any exceptions to this rule were described as a result of long geographic distances and/or a lack of personnel resources. However, as the discussions unfolded, more nuanced images were constructed through negotiations, as demonstrated in the following discussion:

1–4: I’m thinking… aren’t there some areas in the municipality…

1–3: What?

1–4: I’m just thinking… isn’t there something geographic…

1–8: Something cultural?

1–4: Yes. Some places… In some places they want more help than in others… For example, there is one route we visit only once a week.

1–1: Yes, *there* it’s like that. We have discussed…

1–4: Whereas other routes we visit every day.

1–1: Yes

Several: mmmm… yes

1–1: Those who live in [the Sami community] try to manage on their own as long as possible. The neighbours help. They are, as we say in Sami, self-supported. “You ought to be self-supported. You should not get help from others”.

Throughout this short exchange, participants negotiated their initial description of the home care services as being equally available to all inhabitants but gradually realised that they visited parts of the district less often than others. Moreover, they concluded that the reason for their less frequent visits was that those who lived in those parts preferred to manage on their own. In other words, it was constructed as a question of demand rather than supply. In all the focus group interviews, the reluctance of Sami service users and their families to seek and accept help from public healthcare services was an issue:

2–1: I suppose the Sami don’t ask for much help. That’s my impression. At least, we have not had much contact with the Sami.

Moreover, the notion of the Sami as self-supported was evident in the discussions.

2–1: If I look back, I’ve been in the home care services for several years. I see that several need more help, but they don’t accept the offers. It’s about managing on their own. They are used to managing on their own.

“To manage on one’s own” and “to take care of one’s own” did not seem to imply managing without any kind of help. The “own” that the healthcare providers referred to appeared to be a collective “own” rather than an individual “own”. In the healthcare professionals’ narrations, “managing on one’s own” referred to the ability to cope with help from the family rather than from professional healthcare services:

4–3: It’s my impression that the families of Sami persons with dementia take care of their own to a greater extent than the rest of the population. Take care of their own without help from the care services.

Similarly, “managing on one’s own” referred to coping with help from the community. Particular communities in the municipalities, all of which had a predominantly Sami population, were described as self-supported:

1–4: I believe, in that area… That’s a community. They have their own grocery store, their own school and their own postal office. They take care of their own in that community.

In three of the four municipalities involved in the study (FG1, FG2 and FG4), healthcare services were localised in the municipality centre but home care services provided to the entire municipality. In the fourth municipality, the healthcare services were localised in two different areas due to geographical conditions. The focus group in this municipality (FG3) consisted of healthcare providers from only one of these locales, an area of the municipality with a predominantly Sami population, and four out of five participants self-identified as Sami. In many respects, this interview differed from the other three interviews. Rather than speaking about the Sami as “them”, they used the terms “we” and “us”.

As in the three other focus groups, reluctance to accept help among Sami service users and their families was evident in this focus group (FG3). However, these discussions did not include experiences with service users’ reluctance to accept help from the local healthcare services, which they represented. Rather, the participants’ narrations focused on the services that were not provided in the local community but in the municipality centre, which was a predominantly Norwegian community:

3–3: The municipality is divided in two. It’s alienating. Even *I* think so. When I come there, it’s like coming to a completely foreign place. We have nothing there. None of the elders want to go to the nursing home there.

Moreover, their narrations addressed the reluctance to accept help from non-Sami healthcare providers:

3–1: If there is a Sami user, and if the families need help, they contact one of the Sami caregivers rather than a Norwegian. That’s natural. You don’t speak to anyone about everything. You pick – yes, you do. We see that.

It is noteworthy that similar to the other three focus groups, self-support was a significant theme in this focus group, albeit from the perspective of persons who actively positioned themselves as insiders. In their narrations, the boundaries between their positions as formal (professional) and informal caregivers were blurred:

3–5: Let’s say that my friend’s grandmother… When I’m in the home care services and there is my friend’s grandmother or an old aunt. Then I’m there doing my job. But after work, I can go back. And then I’m private. Then I’m there to help. Sleep there at night… Make arrangements. ‘I’ll be here tonight and then you can take over’. Even if the relatives are there. They are not always… But they need help and rest.

However, this blurring of boundaries was restricted to healthcare professionals who were related to Sami service users:

3–3: I must say we are not like that among others. We are like that among people we know well, relatives, close family, maybe an aunt, an uncle or a grandmother. We don’t go anywhere. We don’t. Well, if people want us to come, we do. But I couldn’t make myself go to… We aren’t that acquainted with… what should I say… with Norwegians. This is among the Sami. We do this among the Sami.

The active use of terms such as “we” (about the Sami) and “others” (about Norwegians) and the emphasis on the blurring of boundaries between professional and informal caregiving substantiated the image of Sami “taking care of their own”.

## Attributions to socio-historical contexts

In all of the focus group discussions, reluctance to seek and accept help was attributed to broader socio-historical contexts. References were made to the history of assimilation and discrimination of the Sami. These references were made regardless of the participants’ ethnic self-identification, which indicates awareness of the possible impacts of these processes among both Sami and non-Sami healthcare professionals. In the following excerpt, a non-Sami participant related “thresholds for seeking help” to the Sami population’s feelings of inferiority:

4–3: Obviously, historically, the relationship between those who lived by the fjord and those who lived in [the Sami area] has been full of conflicts. The [term referring to the Sami population] has felt that they were not prioritised. Looked down on, maybe. Guess it’s their Sami background. I have too little knowledge about history, but there have been conflicts. A few decades ago. And I guess, particularly among the elderly, some of it remains. The thresholds for seeking help down here have been high. And that may have resulted in them trying to take care of each other rather than contacting the public services.

Despite acknowledging the possible impacts of historical processes, the participant presented these impacts as something “felt” or experienced by the Sami rather than reality. The use of words such as “felt”, “maybe” and “I guess” and the situating of these issues as something in the past (“A few decades ago”) substantiated this impression. In the following exchange between Sami participants, the references to the broader socio-historical context served a slightly different purpose:

3–1: The elderly generation, they are marked by the old… There was a difference between the Sami and the Norwegians […] The Sami were the lowest, and the Norwegians were above. And you can still see that among the elders. If they know that a Norwegian is coming to visit, they dress up and fix their hair. But when a Sami is visiting…

[laughter]

Interviewer: Have you experienced that?

Several: Yes.

3–3: [You can] be yourself.

3–1: That’s how it is! That generation is accustomed to that. We see a lot of that in everyday life. OK, now it’s the 2000s. It won’t be like that when we reach that age. Then things will be normalized. But that generation, they still believe that they are below. They are careful about that. But if Sami come to visit, we laugh and joke and walk around in a bathrobe. Doesn’t matter if the hair is straight up. Small things like that.

As in the first excerpt, the participants attributed the Sami service users’ reluctance to seek and accept help to the history of assimilation and discrimination. However, in the latter exchange, the healthcare professionals positioned themselves as insiders, as Sami. Although they acknowledged that there were differences between the current generation of elders and their own generation (“It won’t be like that when we reach that age”), they rhetorically constituted a community with former generations through the use of “we” (“But if Sami come to visit, we laugh and joke and walk around in a bathrobe”). In this manner, they discuss reluctance not as a matter of not wanting help from public care services *per se* but as a matter of preferring Sami healthcare professionals.

## Attributions to culture

In all of the focus groups, self-support and reluctance to seek and accept help among Sami service users was constituted as a cultural phenomenon.

4–3: I do believe that it’s more in their culture to take care of each other and take care of their own. And the thresholds for contacting the care services are higher.

In other words, the Sami culture itself was considered a barrier to help seeking. Nevertheless, the interview material contains few examples of “Sami culture” in more concrete terms. One peculiar example from one focus group was the assumption that the Sami have lower hygienic standards than the majority population:

2–2: Also, it could be that there are different standards.

Interviewer: Yes?

2–2: … compared to what we are used to. They are not used to having as high hygienic standards, for example. And therefore, they don’t accept as much help as we believe they should have.

2–1: Yes, that might be so…

Another, perhaps less stigmatising, example was a reference to cultural norms about not speaking about illness or difficulties, such as in the following exchange:

2–1: I believe that, generally, the Sami don’t speak much about illness and weakness.

2–2: So I have also heard. They keep those things to themselves.

Although they are very different, the references to hygienic standards and to cultural norms about not speaking about illness and difficulties have common features. Consistent with assumptions about culture as traits, the healthcare professionals attributed reluctance to seek and accept help to the service users’ culture: The service users do not seek help and Sami families and communities are self-supported because it is part of their culture to do so. In contrast, differences among majority service users were identified as individual preferences or traits, as demonstrated in the following exchange:

Interviewer: You mentioned that the Sami population takes care of their own. Have you experienced that people in other families or communities also keep themselves to themselves? Are there others that are difficult to reach?

4–3: I don’t think there are other groups who don’t want help.

4–1: In such cases, it’s more a matter of persons. If they don’t want help, I mean. Yes. And certain diagnoses.

Very few healthcare professionals attributed service users’ reluctance to seek and accept help to the fact that dementia could be particularly difficult to discuss. However, there were a few examples, such as the following:

Interviewer: It has been said that dementia and illness in general is taboo in the Sami society. Do you have any experiences with that?

3–1: Previously…

3–3: In my experience, the Sami don’t complain a lot. Or… I don’t know. I don’t hear much about their nuisances. I don’t know what to say. […] It’s as if they are not used to complaining. […]

3–5: No. Still there is maybe…

3–3: Taboo…

3–5: Shame

3–3: Yes, shame

[…]

3–3: But isn’t it… Earlier it was more of a taboo. They didn’t want to talk about it. But I believe that it’s more open now.

3–5: I hope so. But still, it is sometimes taboo.

In this exchange, the healthcare professional (3–3) initially attributes reluctance to talk about dementia to more general cultural norms about not complaining. However, this statement is nuanced by her colleagues, who acknowledge that dementia may still be associated with shame.

The impact of the healthcare professionals’ cultural background was thematized in several of the interviews. The professionals’ position as Sami was presented almost as an automatic door-opener, such as in the following excerpt:

3–3: Once, I experienced, many years ago. One of the first years, I worked as a LPN in the home care services. There was this Sami couple. I came there in the morning. Then he looked at me and said, “It is so nice when my own… our own come to visit”. It was… Not just the fact that they didn’t have to speak Norwegian. [They could] be themselves. I understood. They could joke and they knew I wasn’t offended. I could say what I wanted. I could say things and know that they weren’t offended. We were more… The chemistry was right between us. I have experienced that, not only that particular time but several other times as well.

Other participants described how they had negotiated their own positions as insiders, such as in the following excerpt:

1–1: When I started in the home care services, I used to have responsibility for that particular route [referring to an area with a predominantly Sami population]. When I introduced myself, I did it the Sami way. I told them about my grandfather and my father, to present myself. And then they knew, and they started talking. It made them feel safe. That’s how it is. When they know my relatives, and whether we are related… And they talk and talk about family and relatives. Mostly, that is positive. I’ve never had negative experiences. Not yet.

Non-Sami participants also acknowledged the impact of the healthcare professionals’ cultural backgrounds on Sami service users’ reluctance to seek help:

4–3: One of the leaders of the care services is a part of… is from… has one foot in the Sami population. I do believe that has meant something for those who have decided to apply for services. That could contribute to a lower threshold for some of them. Lower than if one of us were in that position.

4–2: Absolutely.

## Attributions to language

In all of the focus groups, reluctance to seek and accept help was attributed to language difficulties, such as in the following excerpt:

2–1: Not many of us speak Sami good enough to be able to have good conversations with the service users. That could also be a reason not to accept help.

In some of the participants’ stories, language was identified as a practical and concrete barrier to help seeking:

1–1: I’ve experienced when doing that particular route [referring to a Sami community]… Once, I came to a lady’s house. Then she told me, “The one who came last week… I had to hide the scissors so she wouldn’t cut my bandages. I tried to explain but she didn’t understand”. She couldn’t explain in Norwegian. So she simply hid the scissors to avoid… [laughter]

In other narrations, the significance of language was discussed at a more abstract level. Competence in the Sami language was closely associated with the position as insider and thus contributed to the lowering of barriers for seeking and accepting help, as demonstrated in the following excerpts:

3–3: I want to tell you about one of our service users. He is mentally ill. He told me some years ago. We had a doctor here. And the user said, “I have never told so much about myself to a doctor. Ever”. ‘Cause they communicated in Sami. Both of them. The doctor asked and talked Sami. He felt so safe, he told me. It felt so good being with him, he said. The Sami. So I believe… That proves that it matters.

1–1: The language. You see, among the older generation, language has been very important. If you know the language, you immediately have a relation. Almost. If you know the Sami language you come really close. You feel it immediately. It’s in the air. I can’t explain it but there is something there.

The assumption that Sami language competency provides healthcare professionals with a position as insiders is, however, nuanced in parts of the interview material. In one focus group, Sami participants questioned the benefits of non-Sami colleagues attending courses to learn the Sami language:

3–3: It’s not enough to learn the language. You must know the culture as well.

[…]

3–5: [Non-Sami colleagues] do understand, to some extent. But still, they don’t understand the culture. But they do understand, to some extent.

There were also negotiations of the assumption that Sami-speaking healthcare professionals automatically possess cultural competence:

1–8: Well… I’m concerned with attitudes. Even if you know the Sami language, you don’t necessarily have a cultural understanding. Unfortunately.

Interviewer: Please, explain what you mean by that.

1–8: I’m thinking… Even if you speak the Sami language… I’ve experienced healthcare professionals who have no understanding of the importance of language. Our language is our most important culture carrier. And unfortunately, you meet Sami speakers who don’t understand how important it is to use one’s language, for example. And some lack an understanding of their own culture in encounters with Sami patients. Unfortunately.

1–1: I also believe that it is very…

1–8: So, you have to be very conscious about that.

1–1: I’m thinking… Many healthcare professionals grew up during the assimilation period and they carry… They are not conscious about that. And they wish to be polite.

In this exchange, the healthcare professional (1–8) notes that even healthcare professionals whose mother tongue is Sami may lack understanding of the impact of “their own culture” and the significance of language. This statement, however, is modified by her colleague (1–1) through her attribution to the socio-historical context of assimilation.

In the interview material, there were also narrations that demonstrated that healthcare professionals’ use of the Sami language could increase the barriers to accepting help:

1–1: Once, I came to the assisted living facilities. I knew there was a Sami man there. I visited, and I started to speak Sami. Suddenly he knocked his fist on the table and said, “No! We are in Norway, and in Norway we speak Norwegian! We are not speaking Sami here!” He was from an area where the assimilation policies had a strong grasp. And then he ended up in an assisted living facility here. When they started to speak Sami, he forgot… And I thought, I’ll have to be careful. You can’t just… You end up stepping on… He had closed that door. And maybe that door should remain closed. ‘Cause he… Maybe it was too late for him to do something about that door.

In this narration, the participant demonstrated the importance of knowledge about the socio-historical context and the service users’ personal life stories to use their language competence in sensitive manners.

## Discussion

The public opinion represented in government white papers that suggests the Sami are reluctant to seek help from public healthcare services was evident in the healthcare professionals’ narrations. However, this image was negotiated and nuanced throughout the focus group discussions. The service users’ reluctance to seek and accept help was attributed to macro contexts, such as socio-historical processes and cultural norms, and to micro contexts, such as individual and interpersonal factors including the healthcare professionals’ cultural backgrounds and language competence.

Healthcare professionals who identified as Sami and those who identified as non-Sami attributed the Sami service users’ reluctance to seek and accept help to the assimilation process and experiences of discrimination, to cultural norms about not speaking about illness or difficulties, and to language difficulties. Moreover, the healthcare professionals’ active positioning as insiders or outsiders (Sami or non-Sami) affected their attributions. Several of the Sami healthcare professionals’ narrations demonstrated how their positions as insiders reduced barriers to help seeking among Sami service users. In that sense, reluctance was constituted not as a matter of not wanting help from the public healthcare services *per se* but rather as a matter of preferring Sami (and, even more so, Sami-speaking) healthcare professionals.

Other researchers [24,32] have noted the importance of professionals’ awareness that the history of colonialism, past abuses and violations against indigenous people can complicate the possibilities for seeking and accepting help. Our data indicate that there is an awareness among both Sami and non-Sami healthcare professionals about the possible impacts of the history of assimilation and discrimination against the Sami on their help seeking. However, the healthcare professionals in our study also attributed reluctance to seek and accept help to the service users’ culture. The service users do not seek help and Sami families and communities are self-supported because it is part of their culture to do so. In this manner, culture is constituted as something *they* (the service users) have and as something that affects *their* behaviour. Consequently, the majority culture and its impact on healthcare relations remain unspoken and transparent. In the literature, this mechanism is referred to as *Othering* (see, e.g. [32,33]). Othering also implies the tendency to explain the Sami service users’ preferences as cultural traits shared by all Sami, whereas differences among majority service users are considered individual preferences. In our data, there were examples of both Sami and non-Sami healthcare professionals describing reluctance to seek and accept help as an aspect of the Sami culture.

Notably, there were few examples in the material of attributions of reluctance to seek and accept help to the fact that dementia is a condition that may be particularly difficult to talk about. Although words such as “taboo” and “shame” were used on a few occasions, the participants more frequently referred to Sami cultural norms of not speaking about illness and difficulties in general. The notion that “Sami don’t speak about illness” has been noted in previous research [34]. Paradoxically, the interview material contains multiple narrations demonstrating the opposite. In fact, several of the narrations demonstrated a great willingness among the service users to speak about their difficulties. However, in all these stories, the healthcare professionals involved were constituted as insiders (Sami), such as in the story about the user who had “never told so much about [himself] to a doctor” (3–3) and the story about the nurse who introduced herself “the Sami way” before the service users “started talking” (1–1). In other words, it seems to be less a matter of not speaking about illness and difficulties than a matter of to whom one speaks about such matters. As stated by one of the participants (3–1), “You don’t speak to anyone about everything”. Sami service users’ preferences for Sami healthcare professionals could be conceptualised solely as a matter of cultural and/or language matching. However, the healthcare professionals’ narrations indicated that experiences of Norwegian dominance and Sami inferiority have an impact on healthcare encounters. These statements indicate that “having information in a form or in a language that renders possible active involvement in the design of the services” [9, p. 50] and encountering service users “with the necessary respect for their values and preferences” [9, p. 50] is insufficient to reduce the barriers to help seeking. Healthcare professionals’ awareness of the way that present encounters in healthcare settings are framed and shaped by service users’ previous and prevailing experiences of marginalization and subordination is crucial to avoid omissions or neglect resulting from assumptions about cultural preferences, such as “Sami don’t speak about illness”.

The ILO Convention No. 169 stated the importance of community-based healthcare services and the training and employment of local community healthcare workers [7]. However, in Norway, the tendency seems to be moving in the opposite direction. Throughout several reform processes, local retirement homes have been closed [35,36], and nursing homes and assisted living facilities have been localised in municipality centres. This was also the case for several of the municipalities included in our study. The government has initiated a process of fusing smaller municipalities into larger units, the so-called local government reform (Kommunereformen). Furthermore, inter-municipal collaborations have been suggested as solutions to challenges arising from “the fact that many Norwegian municipalities are very small and lack sufficient resources and competence” [37]. In many municipalities, the Sami communities are typically localised outside the municipality centres. Thus, the tendency to centralise healthcare services could increase the barriers to help seeking among Sami service users. In the years to come, it will be necessary to monitor the impact of inter-municipal collaborations and the merging of municipalities on the use and non-use of healthcare services among the Sami population. It maybe argued that these reform processes are required to secure “sufficient resources and competence”. However, our study demonstrates that the competence required in encounters with Sami service users also involves knowledge about the possible impact of socio-historical processes and cultural norms and the local knowledge needed to make judgments regarding when, to whom and in which contexts to use the Sami language. The latter finding concurs with previous research that has demonstrated the complexity of providing language-appropriate healthcare services in other healthcare contexts [38].

The Norwegian government (along with the governments of all comparable countries) has a policy aiming at older adults aging at home [9]. Home care services are essential for the achievement of this goal, and the need for home care services increases with age and illnesses such as dementia [39]. For older Sami, moving to a nursing home often implies moving away from local (Sami) communities to institutions situated in municipality centres with predominantly Norwegian populations. This may increase the barriers to help seeking, as indicated in the following statements: “The municipality is divided in two. It’s alienating. […] None of the elders want to go to the nursing home there” (3–3) and “The thresholds for seeking help down here [municipality centre] have been high” (4–3). However, our study suggests that aging at home within one’s own community does not necessarily reduce the barriers to help seeking among Sami service users when home care services are administered from municipality centres and the services are provided by healthcare professionals who are considered as outsiders by both themselves and the service users.

## Concluding remarks

Although previous research has not supported the assumption that the Sami use healthcare services to a lesser extent than the majority population, this study demonstrates that healthcare professionals in local healthcare services have experienced reluctance to seek and accept help among Sami service users. Although further research is necessary to examine Sami service users’ own experiences, it is important to consider these issues from the perspective of healthcare professionals. Local healthcare professionals are at the front line of providing care services. The established opinion that Sami “take care of their own” and are reluctant to seek and accept help may lead to omissions or neglect. If self-support is framed as a cultural custom, it may increase the barriers to healthcare professionals’ offers of help. Browne [32] noted, “Assuming an individual’s manner of interacting is necessarily a function of his or her cultural customs overlooks the significance of the ‘burden of history’ shaping everyday interactions and experiences” (p. 2169). Throughout the focus group discussions, the healthcare professionals in our study both maintained and challenged the established opinion that “Sami take care of their own” through attributions to macro and micro contexts, such as the history of assimilation and discrimination of the Sami and the significance of the Sami language in everyday interactions. In this respect, discussions such as those in the focus groups should be encouraged in everyday care practices to challenge stereotyped and simplified explanations of Sami service users’ needs for care services and to increase awareness about how such explanations can influence healthcare and contribute to marginalising practices.

Although the healthcare professionals’ attributions to multiple contexts are promising, the active positioning of “us” and “them” performed by both Sami and non-Sami healthcare professionals is problematic. Discursively shaped boundaries and differences between groups may create the impression that the distance between the groups is too wide to traverse, which in turn may lead to further marginalisation of service users in everyday healthcare encounters.
